# Aspects of Grit Among Dementia Family Caregivers

**DOI:** 10.1093/geroni/igab046.029

**Published:** 2021-12-17

**Authors:** Jyoti Savla, Karen Roberto, Jennifer Margrett

## Abstract

Dementia family caregivers often show deep devotion and a strong sense of purpose and duty toward their relatives needing care. The concept of grit, which includes aspects of commitment, purpose, perseverance, and resilience, is a novel theoretical approach to expanding understanding of dementia family caregiver strengths amidst the challenges they face. Multiple psychosocial and contextual factors are likely to interact with and influence grit among these caregivers. This symposium addresses commitment to the family dementia caregiver role, willingness to embrace the stressful work of caregiving, and perseverance in finding ways to sustain caregiving roles under typical and adverse circumstances. Blieszner focuses on associations between grit and stressors and strains that challenge caregiver well-being and jeopardize continued caregiving. Wilks considers the impact of spiritual support on sustaining resilience among Caucasian and African American dementia caregivers. McCann explores caregivers’ responses to changes in informal support and social interactions available to assist with home care tasks over the course of the COVID-19 pandemic. Albers examines the strengths and resources caregivers draw upon to manage the challenges of caring for and supporting a relative in long-term residential care during the pandemic. Collectively, these presentations provide new insights into the range of influences on aspects of grit and circumstances in which grit sustains caregiving. Discussant Margrett considers the value of the concept of grit for furthering understanding of caregivers’ abilities to manage typical and unique challenges in their caregiver roles and offers suggestions for future research and interventions to enhance grit among dementia family caregivers.

